# Hypocalciuria as a Key to Diagnosis: A Rare Case of GNA11-Related Familial Hypocalciuric Hypercalcemia (FHH) Type 2

**DOI:** 10.7759/cureus.108848

**Published:** 2026-05-14

**Authors:** Thushara Nayani, Prasun Deb, Sandeep Devireddy, Smitha Nalla, Kiran Choudhary

**Affiliations:** 1 Department of Endocrinology, Krishna Institute of Medical Sciences (KIMS) Hospital, Hyderabad, IND

**Keywords:** calcium-sensing receptor (casr), cinacalcet, familial hypocalciuric hypercalcemia, fhh type 2, fractional excretion of calcium, gna11 mutation, hypocalciuria, primary hyperparathyroidism

## Abstract

Familial hypocalciuric hypercalcemia (FHH) is a rare autosomal dominant disorder of calcium homeostasis characterized by mild hypercalcemia, non-suppressed parathyroid hormone (PTH), and hypocalciuria. FHH type 2 (FHH2), caused by mutations in the GNA11 gene, is particularly rare. Differentiating FHH from primary hyperparathyroidism (PHPT) is crucial to avoid unnecessary surgical intervention. We report a 63-year-old male with persistent hypercalcemia and inappropriately normal PTH levels. Biochemical evaluation revealed marked hypocalciuria with a fractional excretion of calcium of 0.0039. Imaging studies were negative for parathyroid adenoma. Whole-exome sequencing identified a heterozygous missense mutation in GNA11 (c.161C>T; p.Thr54Met), confirming FHH2. The patient was treated with cinacalcet 30 mg daily due to significant hypercalcemia calcium levels > 1 mg/dL above the upper limit. At three months, serum calcium decreased, and PTH normalized. This case underscores the importance of urinary calcium assessment and genetic testing in PTH-dependent hypercalcemia and highlights a potential therapeutic role for cinacalcet in selected FHH2 patients.

## Introduction

Calcium homeostasis is maintained through a tightly regulated interaction between the parathyroid glands, kidneys, bone, and gastrointestinal tract. The calcium-sensing receptor (CaSR), a G-protein-coupled receptor expressed predominantly in parathyroid chief cells and renal tubular epithelium, plays a central role in sensing extracellular calcium concentrations and modulating PTH secretion and renal calcium reabsorption [1,2].

Familial hypocalciuric hypercalcemia (FHH) is typically benign and often asymptomatic, but its biochemical profile frequently overlaps with that of primary hyperparathyroidism (PHPT), posing a diagnostic challenge [3,4]. FHH2 is particularly rare and results from impaired intracellular signalling downstream of CaSR activation. The GNA11 gene encodes the Gα11 subunit, which is essential for transducing CaSR-mediated signals via the phospholipase C-IP3-DAG pathway [5,6]. Given the clinical and biochemical overlap with PHPT, careful evaluation is required to avoid misdiagnosis and unnecessary parathyroid surgery. This case illustrates the importance of integrating urinary calcium indices and genetic testing in establishing the diagnosis of FHH2 and highlights the role of cinacalcet in selected patients.

## Case presentation

A 63-year-old male presented with generalized weakness, diffuse body pains, lower back discomfort, and intermittent left flank pain. He had a history of type 2 diabetes mellitus for five years, managed with oral antidiabetic drugs. There was no history of nephrolithiasis, fragility fractures, proximal muscle weakness, or neuropsychiatric symptoms, without a significant family history. On examination, he was hemodynamically stable with a pulse rate of 88 bpm and a blood pressure of 130/80 mmHg. General and systemic examination, including neurological assessment, was normal. Laboratory investigations revealed persistent hypercalcemia (11.6-12.2 mg/dL) with elevated ionized calcium and inappropriately normal-to-mildly elevated parathyroid hormone (PTH) levels, suggestive of PTH-dependent hypercalcemia (details of the investigations in Tables 1-2).

**Table 1:** Routine laboratory investigations ALT - Alanine aminotransferase; AST - Aspartate aminotransferase; SGPT - Serum glutamic pyruvic transaminase; SGOT - Serum glutamic oxaloacetic transaminase

Parameter	Result	Reference Range
Hemoglobin	14.6 g/dL	13-17 g/dL (male)
Total Leukocyte Count	8400/mm³	4,000-11,000/mm³
Platelet Count	210,000/mm³	150,000-400,000/mm³
Serum Creatinine	1.01 mg/dL	0.7-1.3 mg/dL
Blood Urea	26 mg/dL	15-40 mg/dL
SGOT (AST)	26 U/L	10-40 U/L
SGPT (ALT)	23 U/L	7-56 U/L
Alkaline Phosphatase (ALP)	74 U/L	44-147 U/L
Sodium	138 mEq/L	135-145 mEq/L
Potassium	4.0 mEq/L	3.5-5.0 mEq/L
Chloride	102 mEq/L	98-106 mEq/L
Fasting Blood Glucose	109 mg/dL	70-100 mg/dL
Postprandial Glucose	181 mg/dL	<140 mg/dL
HbA1c	8.8%	<5.7%
Serum Protein Electrophoresis	No monoclonal peak	No monoclonal band
QTc Interval	441 ms	<440 ms (male)

**Table 2:** Calcium profile and urinary calcium excretion PTH - Parathyroid hormone; vitamin D (25 OH) - 25-Hydroxy vitamin D; PHPT - Primary hyperparathyroidism; FHH - Familial hypocalciuric hypercalcemia

Parameter	Result	Reference Range
Serum Calcium	11.6 mg/dL, 12.2 mg/dL (1 month apart)	8.5-10.5 mg/dL
Ionized Calcium	1.44 mmol/L	1.12-1.32 mmol/L
Serum Albumin	4.0 g/dL	3.5-5.0 g/dL
Serum Phosphorus	3.4 mg/dL	2.5-4.5 mg/dL
Vitamin D (25-OH)	39.8 ng/mL	30-100 ng/mL
Intact PTH	76 pg/mL	10-65 pg/mL
Serum Magnesium	2 mg/dL	1.7-2.3 mg/dL
Spot Urine Calcium	11 mg/dL	Variable (low in FHH)
Spot Urine Creatinine	231 mg/dL	20-320 mg/dL
24-hour Urine Calcium	96 mg/day	100-300 mg/day
24-hour Urine Volume	4500 mL	800-2000 mL/day
Fractional Excretion of Calcium (FeCa)	0.0039	>0.02 (PHPT), <0.01 (FHH)
Serum Calcium (3 months after treatment)	10.9 mg/dL	8.5-10.5 mg/dL
Intact PTH (3 months after treatment)	55 pg/mL	10-65 pg/mL

A key diagnostic feature was hypocalciuria. The 24-hour urinary calcium excretion was 96 mg/day, and the fractional excretion of calcium (FeCa) was markedly reduced at 0.0039. A FeCa < 0.01 is strongly suggestive of FHH, whereas values > 0.02 favor PHPT. Polyuria in this case can be due to uncontrolled diabetes and partly due to mildly elevated calcium levels.

**Figure 1:**
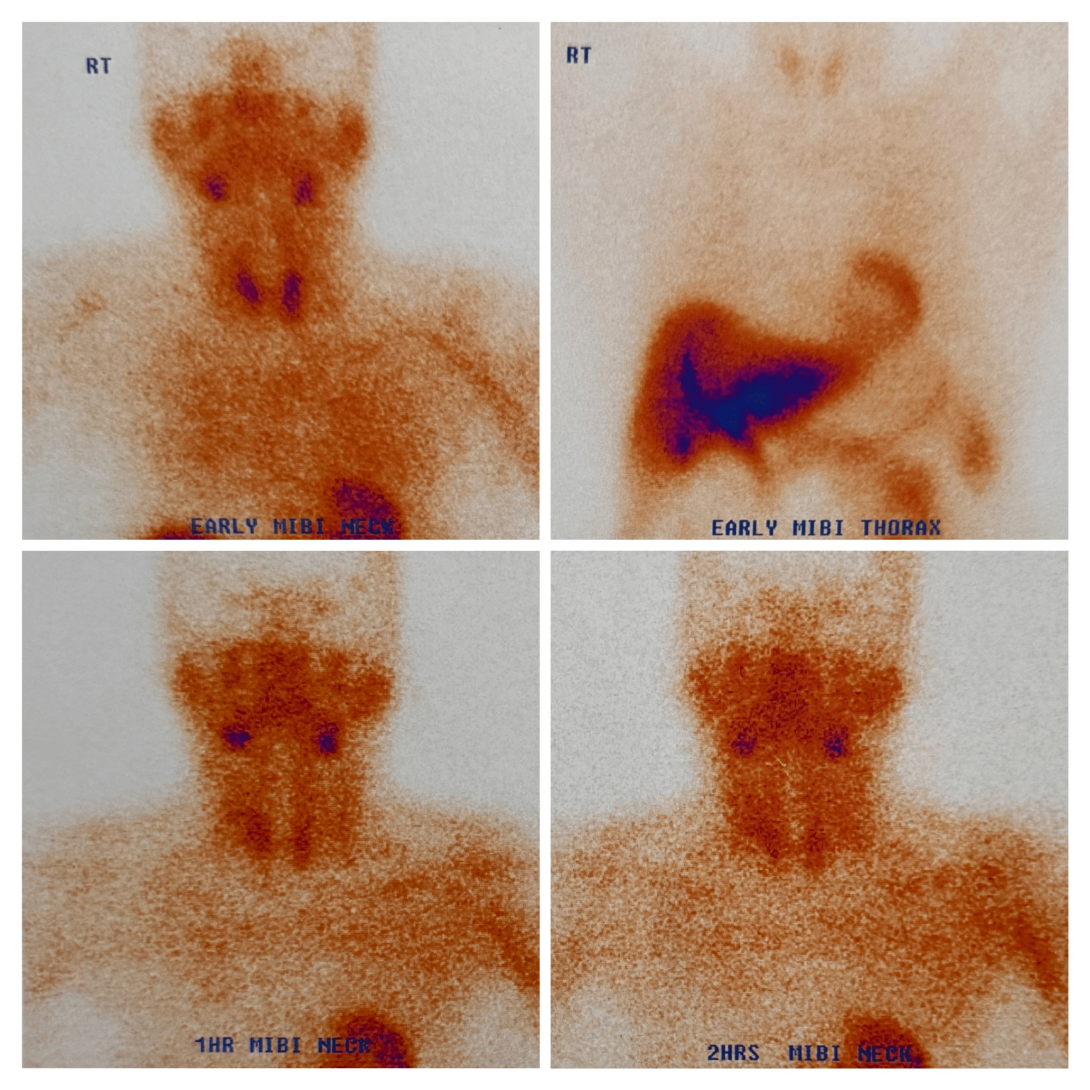
Tc-99m sestamibi scan (early and delayed images) showing physiological tracer uptake in the thyroid and salivary glands, with no focal persistent uptake in the parathyroid region (in the two-hour delayed phase), suggestive of the absence of a parathyroid adenoma

Neck ultrasonography and Technetium-99m sestamibi scintigraphy did not identify any parathyroid adenoma, reducing the likelihood of PHPT (Figure 1).

Dual-energy X-ray absorptiometry revealed osteopenia with T-scores as follows: left forearm: -2.2; AP spine: -1.4; left neck of femur: -1.2; right neck of femur: -1.5; left hip: -0.1; right hip: -0.1; and left forearm: -2.2. Bone mineral density (BMD) was only in the osteopenic range rather than showing overt osteoporosis, which is more characteristic of significant PHPT due to parathyroid adenoma. Given PTH-dependent hypercalcemia with hypocalciuria and negative imaging for parathyroid adenoma, genetic evaluation was performed. Whole-exome sequencing identified a heterozygous missense mutation in the GNA11 gene (c.161C>T; p.Thr54Met), located on chromosome 19p13.3, confirming the diagnosis of FHH type 2.

In view of persistent hypercalcemia exceeding 1 mg/dL above the upper limit of normal, the patient was initiated on Tab. cinacalcet 30 mg once daily. At three-month follow-up, serum calcium decreased to 10.9 mg/dL, and PTH levels normalised. The patient reported symptomatic improvement and continues on therapy with regular follow-up.

## Discussion

FHH is an inherited disorder caused by inactivating mutations affecting components of the CaSR signaling pathway. This results in an increased set-point for calcium-regulated PTH secretion, leading to persistent mild hypercalcemia with relative hypocalciuria [1,3].

FHH is classified into three types: type 1 (CASR mutations), type 2 (GNA11 mutations), and type 3 (AP2S1 mutations), with FHH1 accounting for the majority of cases, while FHH2 is exceedingly rare (<1%) [3-6] (Table 3).

**Table 3:** Classification of familial hypocalciuric hypercalcemia (FHH) Source: [3]

Type	Gene Involved	Protein Affected	Pathophysiology	Clinical Features
FHH Type 1	CASR	Calcium-sensing receptor (CaSR)	Reduced receptor sensitivity to extracellular calcium → impaired suppression of PTH → increased renal calcium reabsorption	Most common (65-80%); usually mild and asymptomatic
FHH Type 2	GNA11	Gα11 (G-protein subunit)	Impaired intracellular signaling downstream of CaSR → decreased cellular response to calcium → increased PTH set-point and renal calcium reabsorption	Rare; clinically similar to FHH1
FHH Type 3	AP2S1	Adaptor protein-2 σ subunit	Defective receptor internalization and signaling → reduced CaSR function → altered calcium sensing	May have higher calcium levels; occasionally more symptomatic

The GNA11 gene encodes the Gα11 protein, which is essential for intracellular signal transduction following CaSR activation. Loss-of-function mutations impair signalling through the phospholipase C pathway, reducing sensitivity to extracellular calcium [6]. In FHH, renal tubular calcium reabsorption is increased, resulting in hypocalciuria. This occurs because the CaSR expressed in the thick ascending limb of the loop of Henle normally inhibits renal calcium reabsorption in response to elevated serum calcium levels. Inactivating mutations in the CaSR signaling pathway, including those involving GNA11, reduce this inhibitory effect, leading to enhanced tubular calcium reabsorption and consequent hypocalciuria [2,7]. A major diagnostic challenge is differentiating FHH from PHPT. Both conditions present with hypercalcemia and non-suppressed PTH levels. However, urinary calcium excretion is a key distinguishing feature. FHH is characterized by hypocalciuria (FeCa < 0.01), whereas PHPT typically shows normal or elevated urinary calcium excretion [8,9]. Imaging studies further help differentiate the two conditions. Parathyroid adenomas are commonly seen in PHPT but are absent in FHH [10]. Genetic testing plays a crucial role in confirming FHH. The identified p.Thr54Met mutation in GNA11 is extremely rare, and only limited cases (around 13) have been reported. Due to this rarity, each additional case contributes significantly to the understanding of genotype-phenotype correlations. We report this case to expand the clinical and therapeutic spectrum of GNA11-related FHH2 [6].

FHH is generally benign and managed conservatively. However, in symptomatic patients or those with significant hypercalcemia > 1 mg/dL above the upper normal limit, pharmacological treatment may be considered [1,3]. Cinacalcet, a calcimimetic, increases the sensitivity of CaSR to extracellular calcium, thereby reducing PTH secretion and serum calcium levels. Although not routinely indicated in FHH, several reports suggest benefit in selected cases [10-13]. In our patient, cinacalcet therapy resulted in a sustained reduction in serum calcium and normalization of PTH over three months. Long-term follow-up is recommended to monitor biochemical parameters and assess for rare complications. Additionally, given the autosomal dominant inheritance, first-degree relatives may benefit from screening and genetic counseling [14].

## Conclusions

This case highlights the importance of considering FHH in patients with PTH-dependent hypercalcemia. Hypocalciuria, particularly a low fractional excretion of calcium, is a key diagnostic clue. Genetic confirmation of a GNA11 mutation established the diagnosis of FHH type 2. Recognition of this condition is essential to avoid unnecessary surgical intervention. Although FHH is usually benign, cinacalcet may be an effective therapeutic option in selected symptomatic patients.
